# Brachial Plexus Injuries in Sport Medicine: Clinical Evaluation, Diagnostic Approaches, Treatment Options, and Rehabilitative Interventions

**DOI:** 10.3390/jfmk5020022

**Published:** 2020-03-30

**Authors:** Immacolata Belviso, Stefano Palermi, Anna Maria Sacco, Veronica Romano, Bruno Corrado, Marcello Zappia, Felice Sirico

**Affiliations:** 1 Department of Public Health, University of Naples “Federico II”, 80131 Naples, Italy; immacolata.belviso@unina.it (I.B.); stefano.palermi@unina.it (S.P.); annamaria.sacco@unina.it (A.M.S.); veronica.romano@unina.it (V.R.); bruno.corrado@unina.it (B.C.); 2 Department of Medicine and Health Sciences, University of Molise, 86100 Campobasso, Italy; marcello.zappia@unimol.it; 3 Musculoskeletal Radiology Unit, Varelli Institute, 80126 Naples, Italy

**Keywords:** brachial plexus, nerve injuries, sport medicine, rehabilitation

## Abstract

The brachial plexus represents a complex anatomical structure in the upper limb. This “network” of peripheral nerves permits the rearrangement of motor efferent fibers, coming from different spinal nerves, in several terminal branches directed to upper limb muscles. Moreover, afferent information coming from different cutaneous regions in upper limb are sorted in different spinal nerves through the brachial plexus. Severe brachial plexus injuries are a rare clinical condition in the general population and in sport medicine, but with dramatic consequences on the motor and sensory functions of the upper limb. In some sports, like martial arts, milder injuries of the brachial plexus can occur, with transient symptoms and with a full recovery. Clinical evaluation represents the cornerstone in the assessment of the athletes with brachial plexus injuries. Electrodiagnostic studies and imaging techniques, like magnetic resonance and high-frequency ultrasound, could be useful to localize the lesion and to define an appropriate treatment and a functional prognosis. Several conservative and surgical techniques could be applied, and multidisciplinary rehabilitative programs could be performed to guide the athlete toward the recovery of the highest functional level, according to the type of injury.

## 1. Introduction

Brachial plexus injuries (BPIs) represent a rare pathology in the general population, with rates of incidence and prevalence difficult to quantify exactly [1,2,3]. Most of these injuries determine relevant motor and sensory impairments of the upper limb, with disability and functional limitations [4]. Their etiology is different and could be related to several direct and indirect causes [5]. According to the anatomical extension of the injury and to the degree of nerve involvement, different therapeutic approaches are needed in order to obtain a clinical restoration of functions, although it is not always possible, and some limitations could be persistent.

The clinical suspicion and assessment of the BPIs, as well as their diagnosis and treatment, are difficult and require a high anatomical and functional knowledge and the cooperation among different specialists with a complex rehabilitation program.

In sport medicine, injuries of brachial plexus are rare, but some of these injuries have dramatic consequences on sport performance and an ending-carrier effect [6,7].

The sport doctor should be aware about the most common potential BPIs related to a specific sport activity. Often, the medical staff engaged in the management of the athletes represents the first source of medical consultation for the athlete, both in competition and during training sessions. Moreover, health personnel involved in sport medicine have to manage acute phase, rehabilitation, and return to sport of the athlete. Beyond severe BPIs, some forms are more common in the athletic population and with a favorable prognosis. The sport doctor should be able to recognize a possible lesion of a peripheral nerve and to guide the athlete towards the most appropriate specialistic management.

Therefore, the aims of the present paper are to review the anatomy of the spinal nerves and of the brachial plexus, to analyze the causes, the patterns, and the degrees of lesion of brachial plexus in sport medicine. Our study aimed also to summarize the key concepts of clinical evaluation and imaging techniques adopted to carry out a definite diagnosis, and, finally, to list the available common conservative, rehabilitative, and surgical options of treatment.

## 2. The Spinal Nerves

A spinal nerve is a mixed nerve containing motor, sensory, and autonomic fibers, originating from the right and left side along the spinal cord [8,9,10].

Through spinal nerves, efferent information is carried from spinal cord to muscles (motor innervation) or to structures under autonomic control (i.e., sweat glands, vessels, arrector pili muscles). Conversely, afferent information is conveyed from periphery to spinal cord, in particular from specific cutaneous (Meissner’s corpuscles, Pacinian corpuscles, Merkel’s disks, free nerve endings, and others) and non-cutaneous receptors (neuromuscular spindles, Golgi tendon organs) to the posterior horn of the spinal cord [11].

**Figure 1:**
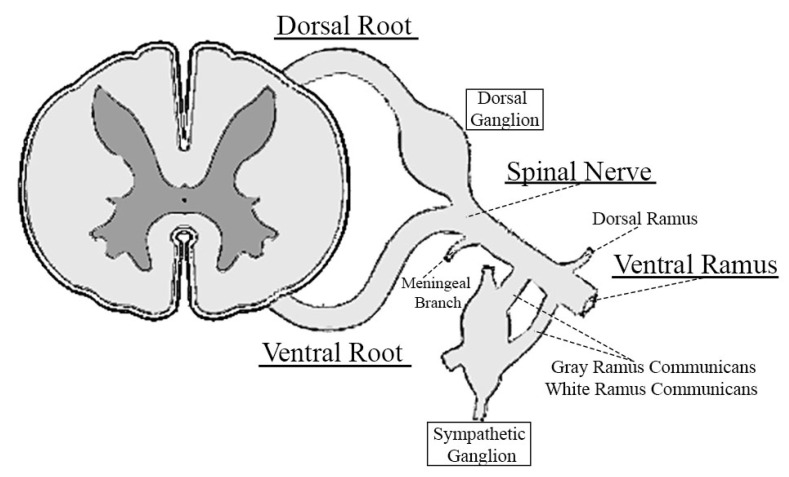
Anatomy of a spinal nerve and its branching pattern.

Each spinal nerve arises from the fusion of a ventral root and a dorsal root. (Figure 1). Ventral motor roots contain axons of motor neurons and the cell bodies of these neurons are located in the anterior horns of the spinal cord. Instead, dorsal sensory roots include axons of sensory neurons and their cell bodies are located outside the spinal cord, in an enlargement of the dorsal root itself (ganglion of the dorsal root), proximal to the fusion between ventral and dorsal roots. When a single ventral root and its correspondent dorsal root joint to form the spinal nerve, it emerges through intervertebral foramina on right and left side of vertebral canal. Just after its emergence, the spinal nerve divides in several branches [12]: (i) a recurrent meningeal branch that re-enters through the intervertebral foramina to innervate dura and spinal vessels, intervertebral joints, intervertebral discus, vertebral periosteum, and other vertebral structures; (ii) a white and grey communicans rami directed to sympathetic paravertebral ganglia (autonomic nervous system); (iii) a dorsal ramus containing motor fibers for axial muscles and sensory fibers for skin of the back (paraspinal region); (iv) a ventral ramus directed to target muscles of thorax, abdomen, and upper and lower limbs.

In some body regions, such as the thorax, the organization of these ventral rami is simple; ventral rami of single spinal nerves, that emerged from individual spinal cord segments, run independently until they reach their specific target structures (i.e., intercostal muscles). Nevertheless, the spinal nerves raised from the cervical region and from the lumbo-sacral region of the spinal cord are directed to the upper and lower limb structures, respectively. Ventral rami of these spinal nerves have a more complex organization: indeed, these ventral rami undergo a rearrangement in an anatomical network of nerves called a plexus [13].

Therefore, a plexus is an anatomical structure interposed between the spinal cord and target structures (muscles and skin of upper and lower limbs) and is made up by the rearrangement of ventral rami of spinal nerves coming from different spinal cord levels. In the cervical region the rearrangement of ventral rami of cervical spinal nerves forms the cervical plexus (C1, C2, C3, and C4) [14] and the brachial plexus (C5, C6, C7, C8, T1) [15].

Hence, each peripheral nerve distal to the plexus contains efferent motor axons from several ventral rami of several spinal nerves, originated by motor neurons located in different spinal cord levels (myelomeres). In the same way, each peripheral nerve conveys afferent information that, through the plexus, is sorted into several ventral rami of various spinal nerves and reaches the spinal cord through several dorsal roots entering at several spinal cord levels (myelomeres).

## 3. Anatomy of the Brachial Plexus

The brachial plexus is a complex anatomical structure involved in the motor innervation of all muscles of upper limb, including torachoappendicular muscles (i.e., pectoralis major), periscapular muscles (i.e., supraspinatus, infraspinatus), arm, forearm, and hand muscles [15]. Sensory cutaneous innervation and autonomic functions of the upper limb are also conveyed through the brachial plexus structures.

Anatomically, the brachial plexus is a nerve network with complex anatomical relationships and with a detailed topography, extending from laterocervical region to distal axilla [16]. Therefore, due to its anatomical extension and position, the brachial plexus has a relationship with neck muscles, clavicle, and axilla encountering other nervous, vascular, lymphoid, and tendinous structures along its course through the cervicoaxillar canal. This complex anatomical topography exposes the brachial plexus to some pathological processes, especially in traumatic accidents.

**Figure 2:**
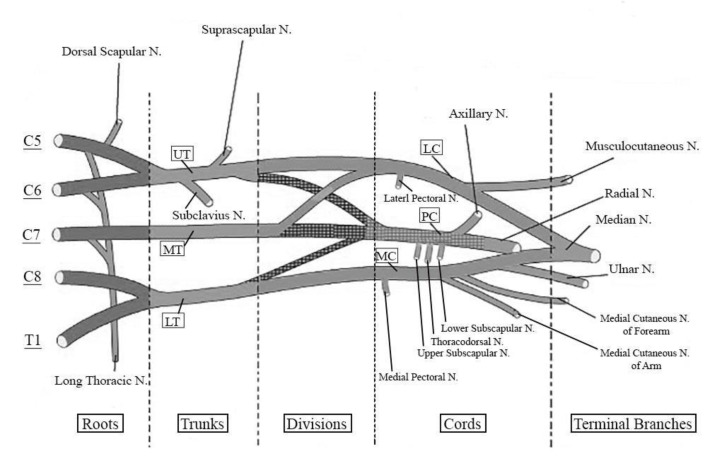
Schematic representation of brachial plexus. UT: Upper Trunk. MT: Middle Trunk. LT: Lower Trunk. LC: Lateral Cord. PC: Posterior Cord. MC: Medial Cord. N: Nerve.

The brachial plexus is a bilateral structure formed by the ventral rami of C5, C6, C7, C8, and T1 spinal nerves (Figure 2) [17]. Along the brachial plexus, ventral rami join and diverge successively defining a network of nerves. Through it, nerve fibers rearrange and each nerve emerging distal to the brachial plexus contain nerve fibers coming from different spinal nerves [18].

The first rearrangement reduces the number of nervous structures from five to three. Indeed, the ventral rami of C5 and C6 join into the Upper Trunk, the ventral ramus of C7 continues alone as the Middle Trunk, while the ventral rami of C8 and T1 join to form the Lower Trunk of the brachial plexus. Therefore, from five ventral rami of five spinal nerves, three trunks are formed (Upper, Middle, and Lower). Distally, each trunk divides in an anterior and a posterior division, passing from three structures (the trunks) to six structures (the divisions). The anterior divisions of Upper and Middle Trunks join to form Lateral Cord. The anterior division of Lower Trunk continues isolated as Medial Cord. The three posterior divisions of the three trunks join to form the Posterior Cord. Therefore, the six divisions (coming from the division of the three trunks) rearrange to form three cords (Lateral, Medial, and Posterior Cord) [19]. Each cord divides in terminal branches directed to shoulder, arm, forearm, and hand muscles. The Lateral Cord divides in two branches: the musculocutaneous nerve and the lateral root of median nerve. The Medial Cord divides in two branches: the ulnar nerve and the medial root of the median nerve. The lateral and medial roots of the median nerve join to form the median nerve. The Posterior Cord divides in two branches: the axillary nerve and the radial nerve.

In summary, the five spinal nerves forming the brachial plexus join to form three trunks, each divide in an anterior and a posterior division. The six divisions rearrange to form three cords: each cord divides in terminal branches [15,16]. Therefore, the musculocutaneous, median, ulnar, radial, and axillary nerves should be considered the terminal branches of the brachial plexus.

Nevertheless, other nerves arise from the brachial plexus without crossing the brachial plexus entirely. Indeed, some nerves arise from ventral rami, from trunks, from divisions, and from cords directly and are not considered “true” terminal branches of the brachial plexus. In a proximo-distal order, two nerves arise from the spinal nerve level (ventral rami): the long thoracic nerve and the dorsal scapular nerve. The long thoracic nerve [20] is formed by the fusion of fibers originating from ventral rami of C5, C6, and C7 and it supplies the serratus anterior muscle. The dorsal scapular nerve [21] emerges from the anterior ramus of C5 and innervates elevator scapulae muscle, rhomboid major and rhomboid minor. These nerves branch from the anterior rami of spinal nerves, proximal to trunk formation and without crossing trunks, divisions, or more distal part of the brachial plexus.

Some nerves emerge from trunks directly. In particular, the subclavius nerve and the suprascapular nerve arise from the upper trunk of brachial plexus and carry fibers from C5 and C6 spinal nerves. The subclavius nerve [22] provides innervation to subclavius muscle. The suprascapular nerve [23] provides motor innervation to supraspinatus and infraspinatus muscles. No nerves arise from divisions directly, but several nerves emerge from lateral, medial, and posterior cords. Lateral and medial pectoral nerves provide motor innervation to pectoralis major and pectoralis minor muscles. Lateral pectoral nerve [24] arises from lateral cord and carries fibers coming from C5, C6, and C7 to innervate the clavicular head of pectoralis major muscle. The medial pectoral nerve [24] arises from the medial cord and innervates the sternal head of pectoralis major muscle. In the most common anatomy, the medial pectoral nerve reaches the pectoralis major muscle after having pierced and innervated the pectoralis minor muscle. The other two nerves arise from the medial cord: the medial cutaneous nerve of the arm and the medial cutaneous nerve of the forearm [25]. These nerves, carrying fibers from C8 and T1 spinal nerves, are pure sensory nerves without motor innervation for muscles in the upper limb. They carry afferent signals from medial cutaneous areas of the arm and forearm. Three nerves arise from the posterior cord, proximally to its terminal division in the radial nerve and axillary nerve: upper subscapular nerve, thoracodorsal nerve, and lower subscapular nerve. Upper and lower subscapular nerves [26] are directed to the subscapularis muscle. Moreover, a branch of the lower subscapular nerve [26] provides innervation to teres major muscle too. The thoracodorsal nerve [27] provides motor innervation to latissimus dorsi muscle. Therefore, beyond the terminal branches (musculocutaneous, ulnar, median, radial, and axillary nerves), two nerves arise from spinal nerves level, two nerves arise from trunks level, and seven nerves arise from cords level.

True terminal branches of the brachial plexus innervate all muscles in the arm, forearm, and hand. Only the axillary nerve [28] serves proximal muscles. Indeed, this nerve provides motor innervation to deltoid muscle and to teres minor muscle. The musculocutaneous nerve [29] innervates muscles in the anterior compartment of the arm (biceps brachii, coracobrachialis, and brachialis muscles). The radial nerve [30] innervates muscles in the posterior compartment of the arm: triceps and anconaeus muscles (anconaeus muscle cross the elbow joint and therefore some authors consider it as a muscle of the posterior compartment of the arm, while other authors consider it as muscle of the posterolateral compartment of the forearm). The radial nerve also supplies all muscles in the posterolateral (extensor) compartment of the forearm. Indeed, the radial nerve innervates 11 muscles in this compartment (brachioradialis, extensor carpi radialis longus, extensor carpi radialis brevis, extensor carpi ulnaris, extensor digitorum, extensor digiti minimi, abductor pollicis longus, extensor pollicis brevis, extensor pollicis longus, extensor indicis, supinator). In the forearm, the radial nerve divides in two terminal branches: superficial branch of the radial nerve (pure sensory) and the posterior interosseous nerve (pure motor).

The median nerve and the ulnar nerve do not supply muscles in the shoulder and arm region. The median nerve [31] provides innervation of almost all muscles in the anterior compartment of the forearm (pronator teres, flexor carpi radialis, flexor digitorum superficialis, flexor digitorum profundus (II-III), flexor pollicis longus, palmaris longus, pronator quadratus). After this course, it passes through the carpal tunnel and provides innervation to muscles of the thenar eminence and to first and second lumbricals.

The ulnar nerve [32] supplies only two muscles in the forearm: the flexor carpi ulnaris and the flexor digitorum profundus (IV-V). Conversely, it supplies several muscles in the hand: muscles of the hypothenar eminence, adductor pollicis, deep head of the flexor pollicis brevis, dorsal interossei, ventral interossei, and third and fourth lumbricals.

## 4. Nerve Injuries: Pathophysiology and Impact on Prognosis

In the assessment of a patient with a nerve injury, it is important to evaluate the mechanisms behind the lesion and to establish the pathophysiological mechanism involved.

A peripheral nerve is made up of nervous tissue, axons of afferent and efferent neurons, surrounded by connective tissue [33]. Connective tissue is organized to form endoneurium (around single axon), perineurium (around fascicles), and epineurium (around the nerve). Moreover, although some axons are amyelinic, larger axons directed to skeletal muscles or coming from skin receptors are surrounded by a myelinic sheath, which is able to conduct action potential properly. In a nerve injury, all these anatomical structures could be involved. Their level of involvement is strictly related to the prognosis and guides the choice of treatment.

To this scope, several classifications of nerve injuries have been proposed. Some of them are complex and detailed (i.e., Sunderland classification) [34]. In clinical practice, the Seddon classification [35] is commonly used, with three-degrees of lesion: neuroapraxia, axonotmesis, and neurotmesis.

Neuroapraxia [36] is functional damage of the nerve; axonal structures and connective tissue are intact. Anatomically, damage to the myelin has been proposed as a mechanism. The nerve is not able to conduct the action potential and therefore motor and sensory symptoms are present. Nevertheless, the persistence of the axon still gives trophic stimuli to the muscle and atrophy related to denervation is absent. This lesion is transient and there is a complete recovery of function, within 2 weeks usually. Although simple and with no relevant consequences, this lesion seems to be the most common lesion in nerve injuries, especially in BPIs related to sport activity [37].

In more severe traumas, axonal structures have been damaged [38]. The portion of the axon distal to the site of lesion degenerates (Wallerian degeneration) and its muscle fibers lose innervation and trophic stimuli, determining muscle atrophy related to denervation mechanisms. These “orphan” muscle fibers could be reinnervated mainly through two mechanisms [39]. Axons from the proximal part of the damaged nerve may regrow and regain their muscle fibers (regrowth reinnervation) or adjacent axons can “adopt” orphan muscle fibers through collateral sprouting of axonal terminals within the muscle (collateral reinnervation). Regrowth of the axons is related to the distance from the site of lesion and the target muscle and it is usually a difficult and slow process. Moreover, connective structures have to be intact to allow regrowth and reinnervation. In any case, the functional relevance of the reinnervation processes is difficult to foresee, even depending on the number of axons involved. These kinds of lesions have a difficult prognosis and they should be monitored carefully; they can show sufficient functional spontaneous recovery or poor rehabilitative results.

The most dramatic scenario is represented by neurotmesis [40]. In these lesions, all structures of the nerve are involved (myelin, axon, and connective tissue). Sometimes the nerve should be separated with a gap between the two nerve extremities. A stump neuroma should be present, as tentative regrowth by the nerve. Obviously, motor and sensory fibers are completely interrupted as a result in this case, and all muscle fibers managed by the nerve lose motor innervation and trophic stimuli, determining a severe atrophy and loss of function. In this situation, spontaneous recovery is impossible and surgical treatment is required. These lesions are common in severe traumatic BPIs unfortunately (i.e., cervical roots avulsions).

Therefore, in each patient with a nerve injury it is necessary to define the etiology behind the lesion, localize the lesion carefully, and define the type of lesion. Indeed, this is unavoidably related to treatment and prognosis.

## 5. Epidemiology of BPIs and Their Relevance in Sport Medicine

Injuries of peripheral nerves can have a wide spectrum of etiology. Some diseases could be classified as primary diseases of the peripheral nerve, i.e., hereditary neuropathy [41]. In other cases, systemic diseases could determine peripheral nerve damage, as in metabolic neuropathies [42] (i.e., diabetes, renal insufficiency, amyloidosis, and others). Other systemic causes of neuropathy are toxic [43] (i.e., alcoholic neuropathy), related to nutritional deficiency [44] (i.e., hypovitaminosis) or to specific medical treatments (chemotherapy-induced neuropathy, post-actinic neuropathy) or correlated to some infectious diseases [45] (i.e., HIV, Lyme disease).

Autoimmune mechanisms justify the onset of a large number of neuropathies [46], some with acute onset (i.e., Guillain-Barrè syndrome), and other with chronic manifestations (i.e., CIDP: chronic inflammatory demyelinating polyneuropathies). Some forms could be characterized by sensory symptoms; some others could determine motor sign uniquely (i.e., multifocal motor neuropathy). These diseases are systemic and determine the involvement of several peripheral nerves usually, affecting structures of the brachial plexus also. Specific characteristics of these forms are out of the scope of the present paper, but they should be taken in account during the examination of a patient with signs of involvement of peripheral nerve structures.

Some peripheral nerve injuries could be iatrogenic [47], such as nerve damage during surgical interventions or related to medical procedures. These forms of nerve injuries, in particular those regarding the brachial plexus, are relevant in sport medicine as they are a non-rare complication of several orthopedic treatments like shoulder surgery and shoulder dislocation reduction.

Primary (i.e., neurinoma) or secondary (i.e., brachial plexus involvement in lung tumors) neoplastic involvement of peripheral nerves is possible [48] and should be taken into account according to the clinical history referred by the patient and to the signs collected during the clinical evaluation.

Nevertheless, the involvement of brachial plexus structures due to these systemic and localized diseases is rare. Indeed, in the epidemiology of the etiology of isolated BPIs, traumatic events remain the main cause of injury [49]. Usually, male subjects of working age are involved in traumatic BPIs, with a male/female ratio of 13.3:1 [5].

A closed lesion of the brachial plexus, mainly related to traction of peripheral nerves, represents 93% of traumatic BPIs [50]. The other reasons are gunshots lesions, lacerations, and iatrogenic lesions.

Supraclavicular structures of brachial plexus (i.e., roots and trunks) are more involved (90%). Complete injuries of brachial plexus are quite common during traumatic lesions (53%). Nevertheless, the upper structures are more affected than lower [5].

Motorcycle accidents are the main cause of traumatic BPIs, accounting for 67% of them [5].

In motorbike or cycling accidents, the arm undergoes a sudden and violent traction caused by the fall and usually the higher the velocity the more severe the clinical presentation of the injury [49].

In occupational or sport activities, data from previous studies reported a pooled prevalence of 10% of traumatic BPIs [5]. Some more detailed data about sport activities show an incidence of BPIs among nearly 4% of trauma related to winter sports [51].

Obviously, BPIs are a common injury in contact sports [49] but their exact incidence is difficult to estimate. Indeed, lesions with neuroapraxia, commonly known as “burners and stingers syndrome” are common in these sports and related to impact (i.e., kick in martial arts) on the supraclavicular region. Likely, the complete functional recovery associated to these lesions determines an underestimation of their exact rate of incidence.

A similar concept should be considered is some sports, even in relation to the equipment. In American football, the use of helmets has reduced the number of severe concussions, but it has caused a higher incidence of trauma in the supraclavicular region with the involvement of the brachial plexus [52].

Even in motorcycle accidents, the adoption of helmets permits the management of severe BPIs often requiring a surgical intervention. Probably, the increase of these cases is related to a higher survival rate for the use of the helmet itself [53].

Moreover, a reporting bias should be considered in the epidemiology of BPIs related to sport activities. Indeed, almost all studies reported the incidence and prevalence of traumatic BPIs that required surgical treatment [54]. Several lesions of brachial plexus showed a less dramatic involvement of nerve structures, with neuroapraxia and partial axonotmesis. In these cases, a conservative approach could determine a successful and sometimes complete recovery. The incidence of these lesions is not quantified in published studies and their prevalence could be underestimated.

Some data show that 30–40% [55] of rugby players have suffered a “stinger” or “burner” at least once. In a Canadian football study, a “stinger” was reported by the 26% of players, during the 2010 football season [56] and only 59% of them were referred to medical staff. Moreover, the history of “stinger” is a risk factor to sustain a new “stinger” and a correlation with the years of sport practice was detected too. In an American football study, the lifetime rate of BPIs (“stinger”) was 50.3% [57] and the incidence seems to be related to the role in the team (highest incidence in running backs and defensive linemen).

Therefore, while incidence rates of severe BPIs seem to be low, the incidence of simpler lesions of BPIs, like burners and stingers, depicts a worrying and an unclear scenario in sport medicine [37].

## 6. Clinical Evaluation

### 6.1. Clinical Findings

BPIs should be suspected in each athlete presenting with symptoms of peripheral nerve involvement [58].

As previously stated, most of the BPIs are associated to indirect trauma, with traction of cervical spinal nerves due to forced movements of the arm or of the cervical spine.

Therefore, a clinical examination aiming to exclude an involvement of cervical spinal cord is mandatory. Radiographs should be performed to rule out cervical vertebral involvement [59]. Any sign of weakness or numbness involving lower limbs or territories distal to upper limbs should be evaluated carefully. After having excluded a cervical spinal cord involvement, the evaluation of segmental strength is useful to localize the level of lesion within the plexus [58]. Localization of the lesion should be difficult, even due to the high degree of anatomical variability between, and even within, subjects [60].

Anatomical knowledge could guide the localization of the lesion. Based on the time from the lesion, the visual inspection could demonstrate selective atrophy in specific muscular groups [61]. Indeed, after axonotmesis or neurotmesis, several days are needed to permit muscular atrophy.

Moreover, visual inspection could make visible two relevant signs able to localize lesion: winged scapula and Bernard-Horner syndrome.

Winged scapula [62] could be associated to several nerve injuries. In particular, lesion of three nerves could determine winged scapula: long thoracic nerve, dorsal scapular nerve, and spinal accessory nerve. Some clinical characteristics, out of the scope of the present paper, help in identification of nerves involved among them [63]. Nevertheless, the long thoracic nerve lesion is the most common form. Its involvement should be isolated and related to several diseases. Interestingly, in a patient with BPIs, the presence of a winged scapula due to a lesion of long thoracic nerve should be investigated carefully. Indeed, the long thoracic nerve arises from C5-C6-C7 at roots level and is the most proximal nerve arising from the brachial plexus [20]. Therefore, its clinical deficit is compatible with a very proximal lesion of brachial plexus, usually at roots level, as roots avulsion. Moreover, Bernard-Horner syndrome is characterized by homolateral miosis, ptosis, and enophthalmos [64]. This clinical presentation is common in proximal involvement of the lower portion of the brachial plexus. Indeed, lesion of C8-T1 roots determines the involvement of the sympathetic trunk with lesions of fibers that from the spinal cord in cervical and thoracic regions go back to head and cranial structures [59]. Although several central localizations could justify this syndrome, its occurrence in a patient suffering from BPIs is indicative of involvement of distal structures of the plexus.

Manual muscle testing, grading voluntary recruitment of skeletal muscles from zero (no movement or perceived muscular contraction) to five (normal strength against gravity and full manual resistance) is commonly used to assess strength [59].

The involvement of the upper trunk is common and determines weakness of proximal muscles (i.e., scapular muscles and muscles of shoulder), while hand muscle function is preserved (Erb-Duchenne paralysis) [65]. Conversely, involvement of the lower trunk is associated to a motor deficit of the intrinsic hand muscles, with normal strength in proximal muscles (Klumpke paralysis) [66].

Ventral rami of spinal nerves, trunks, divisions, cords, and terminal branches of the brachial plexus are mixed nerves, including some sensory fibers. Therefore, BPIs are also associated to sensory symptoms in specific cutaneous areas. Sensory symptoms could include neuropathic pain, numbness, hyperalgesia, allodynia, hypoesthesia, and anesthesia, according to the time from the lesion and to the type of lesion [67]. A high variability of the sensory cutaneous pattern of innervation exists. However, the cutaneous sensory involvement has to be consistent to the pattern of weakness to permit an exact localization of the lesion. It is useful to specify that sensory cutaneous innervation of brachial plexus structures is different from dermatomeric innervation. Indeed, the dermatome is the cutaneous area innervated by a specific dorsal root of a specific spinal nerve. On the other hand, the lesion of a brachial plexus structure implies the involvement of cutaneous regions served by specific fibers of different spinal nerves travelling in the same structure of the brachial plexus [68].

The osteotendinous reflexes appear reduced or absent, according to the anatomical distribution [59]. For example, the bicipital reflex is conveyed through musculocutaneous nerve, lateral cord, upper trunk, and C6 root [69]. Each lesion along this pathway could determine the abolition of the reflex. In addition, changes in the deep tendon reflexes have to be consistent with the patterns of weakness and sensory involvement.

A detailed clinical examination should be sufficient to localize the lesion in most of cases and to orient the following steps in the management of patients suffering from BPIs.

Electrodiagnostic studies and imaging techniques are useful to confirm the diagnosis, to better characterize the lesion, to define the prognosis, and to guide the treatment.

### 6.2. Electrodiagnostic Studies

In BPIs, electrodiagnostic studies include electroneurography and needle electromyography, commonly [70]. Evoked potentials are not useful as routine examinations.

Electroneurography [71] permits the analysis of compound motor action potentials (CMAPs) and of sensory action potentials (SAPs) through electrical stimulation of peripheral nerves and surface recording from muscles or skin areas, respectively. Motor and sensory conduction velocities can be measured. The main rule of electroneurography in BPIs is to confirm a pathological involvement of SAPs [68]. Indeed, due to the anatomical localization of cell bodies of sensory neurons (ganglion of dorsal root), the persistence of normal sensory action potentials is non-compatible with a plexus lesion (SAPs remain normal in patients with BPIs only if an avulsion of dorsal root is present: pre-ganglionic lesion). Conversely, any damage along the nerve reduces the amplitude of sensory action potentials (post-ganglionic lesions). Moreover, the analysis of CMAPs permits restriction of the possible localization of the lesion and exclusion other diseases.

Motor conduction blocks are the electroneurographic manifestations of myelin dysfunction and represent the main finding in neuroapraxial lesions.

Needle electromyography is sensitive in detecting denervation [72]. Therefore, it is useful to demonstrate axonal damage. Hence, it is necessary to demonstrate axonotmesis and neurotmesis. Moreover, through electromyography, it is possible to investigate active recruitment of the muscle and to investigate the morphology of motor unit potentials (MUPs) [59]. Indeed, MUPs analysis could also demonstrate reinnervation progression, as well as it can add information about the prognosis.

Thus, electrodiagnostic studies are able to localize the lesion and to provide functional information; moreover, they permit definition of the type of lesion and follow up of the reinnervation process [59,73]. Unfortunately, they require a specific timing that has to be taken into account. Conduction blocks are immediately detectable through motor conduction studies. After axonal lesions, reduction of CMAPs and SAPs amplitude requires several days. During the first days, the amplitude of the potentials recorded though electrical stimulation distally to the lesion site is still normal and decrement begins according to Wallerian degeneration. CMAPs nadir is reached in 4–5 days, while SAPs nadir requires 7–8 days.

According to the time from the lesion and to the distance of the target muscles, denervation signs are detectable with needle electromyography after several days and usually requires 2–3 weeks to became evident in more distal muscles (21 days) [74].

### 6.3. Imaging

**Figure 3:**
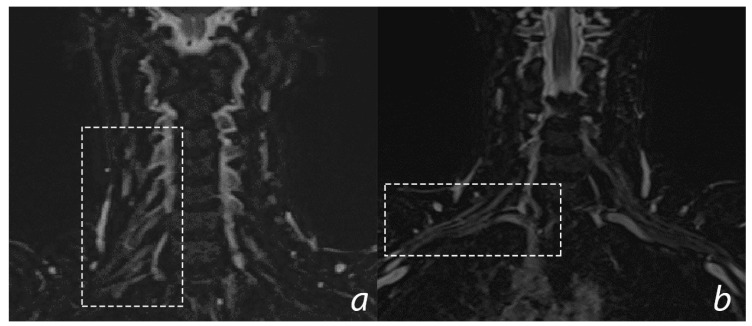
3D T2 STIR SPACE sequence—coronal images of normal Brachial Plexus. (**a**) Dotted rectangle includes cervical spinal nerves emerging from the intervertebral foramina in the supraclavicular laterocervical region; (**b**) dotted rectangle includes the brachial plexus structures passing through the cervicoaxillary canal.

Imaging techniques to investigate peripheral nerves have become more common in the last few years. Indeed, improvements in the equipment allow better investigation of these structures. X-ray and MRI of the cervical spine are commonly performed and are useful to rule out vertebral fractures, spinal cord involvement, and to assess some indexes like the Mean Subaxial Cervical Space Available for the Cord (MSCSAC): the antero-posterior diameter of spinal canal minus the antero-posterior diameter of spinal cord, measured in a sagittal plane. Indeed, some previous results showed an association between risk of BPIs and cervical canal stenosis assessed through this method in athletes [75]. Modern magnetic resonance (MRI) is useful to define the nerves and their relationship with normal or pathological surrounding structures [76]. Nowadays, MRI could provide visualization and investigation of brachial plexus structures directly and could give some information about the neurogenic muscular changes, like denervation (Figure 3).

Regarding ultrasound (US), the diffusion of high-frequencies probes has allowed the exploration of small superficial structures as peripheral nerves. Ultrasounds show some advantages [60]. They are not invasive and allow performing dynamic maneuverers able to investigate stability of peripheral nerves during active and passive movements. US also allows exploration of some parameters of inflammation as vascularization, such as visualizing the ultrastructure of the nerve and the fascicular architecture. Furthermore, US allows undertaking of some measurements like cross-sectional area of the nerve, following the course of several nerves, and, finally, studying their relationship with the other anatomical structures.

**Figure 4:**
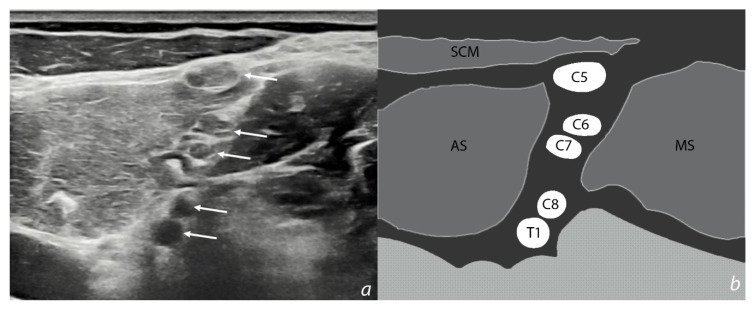
Ultrasound image of the interscalene groove in supraclavicular laterocervical region. Cervical spinal nerves (ventral rami) forming the brachial plexus pass through the anatomical space between anterior and middle scalene muscles, before they merge to form the upper, middle, and lower trunks. (**a**) Ultrasound image. White arrows indicate cervical spinal nerves (ventral rami); (**b**) schematic representation of the interscalene groove. C5 is the most superficial cervical spinal nerve, immediately below the sternocleidomastoid muscle. C5: cervical spinal nerve 5. C6: cervical spinal nerve 6. C7: cervical spinal nerve 7. C8: cervical spinal nerve 8. T1: thoracic spinal nerve 1. SCM: sternocleidomastoid muscle, AS: anterior scalene muscle, MS: middle scalene muscle.

Nevertheless, due to the anatomical complexity of the region, imaging investigation of BPIs is difficult and a high-degree of competence is required [58]. Supraclavicular portions of the brachial plexus could be investigated in lateral neck regions. Spinal nerves could be identified and followed until trunk formation. Some anatomical landmarks, such as anterior and posterior tuberculi of transverse processes of cervical vertebrae, and scalenus muscles (anterior and middle), could be useful in order to localize these structures [77] (Figure 4).

Divisions are not commonly explorable as they are located behind the clavicle and their ultrasound exploration is impossible. Cords and terminal branches are explorable distally to the clavicle and they are commonly visualized through an axillary approach. A relevant anatomical vascular reference, in this case, is due to their relationship with the axillary artery. Even US could give qualitative information about muscles, identifying denervated muscles in which the contractile tissue is substituted with connective and adipose tissues after denervation [77].

Although functional information is not obtained through imaging studies or should be considered “indirect” (i.e., denervation in a muscle could indicate axonal damage of a specific nerve), numerous anatomical and morphological information is provided.

Therefore, in most cases, electrodiagnostic studies and imaging techniques are useful to collect numerous morphological and functional information about the BPIs.

## 7. Therapeutic Options

Therapeutic options are defined according to the type of lesion [61]. Neuroapraxia requires conservative treatment, and the recovery is complete. Neurotmesis requires early surgical treatment. Axonotmesis requires a conservative approach and an accurate follow up to evaluate the reinnervation process. In the case of insufficient restoration of the function, surgical treatment could be required.

### 7.1. Rehabilitation

The main goals or rehabilitation are (i) protection of the injured area, avoiding other damage of nerve structures, (ii) pain control and management of sensory symptoms, (iii) limitation of muscular atrophy, (iv) improvement of strength, (v) restoration of function.

In order to obtain these goals, several techniques are adopted. It has been demonstrated that stretching maneuvers are able to maintain flexibility of skeletal muscles [78]. Moreover, molecular evidence shows that passive stretching promotes the stimulation of mechanoreceptors and slow down the degradation process of proteins, representing the molecular basis of atrophy [79]. Stretching is also relevant to maintain a correct range of motion of the joints functionally correlated to the involved muscles.

Electrical stimulation determines the passive contraction of the denervated muscles. Several types of electrical stimulation have been proposed [80]. Morphology of the waves, duration of electrical stimulation, and frequency of stimulation and position of the electrodes are parameters that should be considered in the treatment protocol. Nevertheless, no evidence supports a specific protocol of intervention about electrical stimulation and several approaches exist [3].

Transcutaneous nerve electrical stimulation (TENS) is commonly adopted to interfere with sensory perception and exerts an antalgic effect [81].

In partial axonal lesions, the active recruitment of the involved muscles could be useful to promote reinnervation and to increase strength and hypertrophy of survival motor units. This goal could be achieved through several rehabilitative techniques (i.e., neuromuscular proprioceptive facilitations [82]). It has been proposed that exercises against external resistance increase the strength of the muscles, through isometric, isotonic, and auxotonique contractions (elastic resistance) [83].

The education of the patient is also important, especially if a relevant sensory involvement is present. The deafferentation of skin tissue is the main cause of skin lesions (cuts, burns, and others) that could facilitate other diseases like infections and orthopedic traumas [84]. Rehabilitative approaches are important to maintain proprioception of deafferented structures also, limiting the impact on central motor representation and control. Some orthoses are useful to facilitate movement or to permit strengthening of specific muscle groups, like hand muscles.

Some BPIs could be very painful and pain management is a difficult topic. Some drugs, like opioids, should be used in neuropathic pains. Some antiepileptic drugs, like pregabalin and gabapentin [85], have shown positive effects in post-traumatic neuropathic pain relief, although some side effects are reported (i.e., dizziness) and discontinuation of treatment is frequent. As in other musculoskeletal conditions [86], some multivitamin supplements and neurotrophic agents are commonly adopted, especially in neuroapraxia and axonotmesis, even if their effectiveness is debated and even if they are commonly used in sports population behind nerve injuries [87]. Perineural steroid and anesthetic injections at the site of nerve injury, commonly adopted for short relief of the pain in other several musculoskeletal conditions [88], are not routinely performed in the management of neuropathic pain but they have shown some positive results [89].

### 7.2. Surgery

The timing of surgical treatment has been extensively discussed in scientific literature [36,54]. Some BPIs (i.e., gunshots injuries) are associated to vascular injuries or open lesions (i.e., cut lesions) and require acute surgical treatment [90]. Neurorrhaphy is often performed, and the anatomical continuity of the peripheral nerve is restored. In case of a significant damage of neural tissue, a simple neurorrhaphy cannot be performed and a nerve graft is required. Usually, the sural nerve (a pure sensory nerve of the leg) is used as donor.

Preganglionic lesions, like roots avulsions, have no spontaneous recovery and a delayed surgical treatment is not useful. Therefore, different surgical approaches are performed as soon as possible [90].

In the large part of BPIs, the mechanism of lesion is mainly due to excessive traction along the peripheral nerve with axonal damage and a variable amount of neuroapraxia is commonly present [91]. In this case, the surgical approach is often delayed for 3–4 months. During this period, diagnostic procedures and rehabilitative treatments were carried out: clinical evaluations and electrodiagnostic studies could be useful to attest progression in recovery of denervated muscles. In the case of poor restoration of function, surgical exploration of brachial plexus structures is required.

Sometimes, it is difficult to restore all functions of muscles innervated by damaged nerves. Therefore, a “strategy” of priority in recovery is necessary. Restoring the function of elbow flexors and shoulder stabilizers is often considered as priority [91]. Several peripheral nerve procedures have been proposed during recent years. Neurotization is commonly adopted [92]. This technique consists of a motor nerve transfer. A donor spare motor nerve is transferred and sutured to a damaged nerve. Intra-plexus neurotization provides transfer of a nerve from the brachial plexus in favor of another nerve within the brachial plexus. For example, some motor branches of the radial nerve directed to the long head of triceps are transferred to a damaged axillary nerve to restore the function of deltoid muscle. In other cases, some extra-plexus nerves could be used. As example, the intercostal nerve could be transferred in favor of the musculocutaneous nerve or the spinal accessory nerve could be used to reinnervate muscles supplied by the suprascapular nerve [93]. In some cases, terminal branches of a spared motor nerve could be inserted directly in the target muscles to promote the generation of new neuromuscular junctions.

Some procedures provide the transfer of a specific fasciculus of a nerve, distal to the brachial plexus, in favor of another nerve. For example, in the procedure described by Oberlin et al. [94], the ulnar fasciculus directed to flexor carpis ulnaris is transferred in favor of motor branches for biceps brachii innervation.

Several orthopedic procedures were used in recent years, like shoulder arthrodesis, wrist arthrodesis, or even upper limb amputation. Improvements in surgical peripheral nerve procedures have reduced their utility dramatically.

In specific cases, in which surgery was performed too late, and nerve restoration is not possible, tendon transfers have become a valuable palliative surgical treatment [95]. In these cases, the target of the surgery is the transfer of a tendinous structure of normally innervated muscles. Just as an example, trapezius muscles attachment could be transferred to humerus to restore abduction or external rotation, the pectoralis major’s tendon could be transferred in favor of biceps tendon to restore elbow flexion. The triceps tendon could be transferred in favor of biceps tendon to restore elbow flexion. In the late 70s some authors [96] described a technique of free muscle transfer. Some muscles far from the site of lesion, are transferred to restore some biomechanical functions. For example, gracilis muscle is transferred in favor of elbow flexors. This technique requires microvascular sutures and it is technically difficult.

In the last years, a bionic reconstruction approach has been described in patients with global BPIs including lower root avulsions. These patients have a severe impairment of hand function, and they have received previous surgical brachial plexus reconstruction without significant restoration of distal muscles voluntary contraction. Therefore, they have no other treatment options available. This approach has been conducted in two stages: first useful electromiographic signals for prosthetic control were identified and/or created through assessment of voluntary recruitment of some forearm muscles or by a free muscle transfer of gracilis muscle. After a dedicated rehabilitation program to improve the control of these muscles, an elective amputation of hand has been performed and a replacement with a mechatronic prosthesis was conducted. Although the results are restricted to a limited cohort of patients and this technique is indicated in BPIs without other treatment options, a significant functional improvement has been proved [97,98].

## 8. The Sport Doctor and the Management of the Athlete with BPIs: From the Side-Line to the Return to Play

As previously described, some sport activities are associated to BPIs with different degree of motor and sensory impairment. “Stinger” involving the upper trunk of brachial plexus represents the most common BPIs in athlete. This condition also represents the most common upper extremity neurologic injury in the athlete [99]. They are acutely involved and could have different clinical presentation according to the degree of nerve damage. In the most favorable scenario, some forms of stingers are not reported to physician. This high percentage of “missed” athletes could be justified by the lack of long-lasting symptoms. Indeed, some athletes could experience only self-limiting sharp and burning pain, radiating down the arm with paresthesia and numbness lasting for few seconds to minutes. Therefore, even if the time lost in athletic activities is 2.9 days on average, 79.3% of athletes continue a normal training program after a simple stinger [100]. Muscular strength could be reduced, and weakness can persist for few hours to 6 weeks or more. [101]. As described, superior cervical roots (C5 and C6) and upper trunk are the most affected structures of the brachial plexus. This could be related to several factors such as (i) vulnerability of upper trunk at Erb’s point (supraclavicular region); (ii) direct trauma on nerve structures; (iii) anatomical variability in intervertebral foramina (with smallest at C4-C5 level); (iv) mechanism of injury further reducing the diameter of foramina, like hyperextension with ipsilateral side bending and contralateral cervical spine rotation [99,102,103].

Sideline evaluation of athlete with a suspected BPIs is difficult. In the severe cases, the athlete is assessed during competition and the main clinical problem to rule out is the involvement of cervical spinal cord. The helmet should not be removed and/or a rigid collar should be applied. Bilateral arm signs or involvement of lower limbs are not in agreement with BPIs and should increase the suspicion of a spinal cord involvement.

The less severe athletes are commonly assessed after the competition. Muscular strength of C5-C6 muscles should be assessed, such as deltoid, biceps brachii, supraspinatus, and brachioradialis. These muscles are innervated through different nerves raised from brachial plexus and “share” a unique anatomical connection through the upper trunk. The involvement of serratus anterior muscle (long thoracic nerve) and/or rhomboideus muscles and levator scapulae muscle (dorsal scapular nerve) could help to distinguish between lesion of C5-C6 roots and upper trunk. [59] In the most common anatomy, triceps muscle has to be normal (as innervated through middle trunk containing fibers of root C7) and hand muscles too. Numbness is commonly reported in the deltoid region and on the lateral aspect of the arm and forearm to the thumb. Bicipital and brachioradial reflexes are commonly reduced or absent.

Patients with muscular weakness and with persistent symptoms should be clinically assessed and followed for at least two weeks. Indeed, in BPIs characterized by neuroapraxia, a complete restoration of functions is usually evident during this period.

In case of persistent pain and severe weakness, imaging technique and electrodiagnostic studies should be performed. These data could determine the exact localization of the lesion and are essential to guide the treatment in this phase.

Indeed, in axonotmesis and neurotmesis, needle electromyography is able to demonstrate active denervation. Effective reinnervation processes could be recorded in the recovery phase of axonotmesis. In neurotmesis, like root avulsions, imaging techniques are more useful for demonstrating the anatomical damage and to avoid needless delay in surgical treatment.

In athletes with axonotmesis, the rehabilitation procedures should be undertaken as soon as possible. The clinical evaluation should guide the assessment of the athlete. Indeed, needle electromyography could demonstrate changes for several months after nerve damage and cannot be used as a method to determine the time to return to play [104].

In hyper acute phase, if the athlete presents transient BPIs lasting for a few seconds and with a complete restoration of motor and sensory symptoms, sport activity can be permitted. If athlete complains of persistent symptoms, he/she should be assessed routinely. Return to play is permitted when sensory symptoms are solved, and muscular strength is restored.

It is important to take into consideration the high rate of reoccurrence of stinger, especially in athletes with anatomical abnormalities, like intervertebral foramina stenosis. In these athletes, the signs and symptoms could last longer than usual, and sport activity should be limited. Obviously, in athletes with persistent weakness, intractable pain and severe limitations of cervical spine range of motion, sport activity is prohibited. In severe cases, in which neurotmesis or root avulsion are evident, the treatment is mainly based on a surgical approach.

The choice of the equipment, the type of sport activity performed, the role in the team, and some technical advice are useful strategies of prevention that should be practiced in sports at high-risk of BPIs.

## 9. Conclusions

Severe BPIs are a rare condition in sport medicine. Instead, milder forms of brachial plexus involvement seem more common among athletes, according to the type of sport, the role in the team, and the sporting history, and they should be related to some specific equipment. Severe BPIs are a difficult topic and their management requires a multi-professional team. Clinical investigation is the cornerstone in the definition and localization of injury. Electrodiagnostic studies and imaging techniques provide valuable morphological and functional information able to guide the treatment and to define the prognosis. Rehabilitative approaches should be structured and should include several options of treatment aimed to control pain and to promote the restoration of motor and sensory functions. In most severe cases, specialistic neurosurgical approaches should be performed on peripheral nerves or on tendinous structures to restore functions, which could not be regained conservatively.
